# Evaluation of the Efficacy of Thyroid Imaging Reporting and Data Systems Classification in Risk Stratification and in the Management of Thyroid Swelling by Comparing It With Fine-Needle Aspiration Cytology and Histopathological Examination

**DOI:** 10.7759/cureus.59949

**Published:** 2024-05-09

**Authors:** Abhishek K Saw, Zenith H Kerketta, Khushboo Rani, Krishna Murari, Kritika Srivastava, Ajay Kumar, Sunny LNU, Anish Baxla, Nabu Kumar, Nusrat Noor

**Affiliations:** 1 General Surgery, Rajendra Institute of Medical Sciences, Ranchi, IND; 2 Obstetrics and Gynaecology, Rajendra Institute of Medical Sciences, Ranchi, IND; 3 Trauma Surgery & Critical Care, All India Institute of Medical Sciences, Rishikesh, Rishikesh, IND; 4 General Practice, Clinica Cure Hospital, Ranchi, IND

**Keywords:** biopsy, bethesda scoring, tirads scoring, fine-needle aspiration cytology (fnac), ultrasound, thyroid disorders

## Abstract

Background: Thyroid nodules are a common clinical challenge, with a significant proportion being cancerous. Fine-needle aspiration cytology (FNAC) is widely used for diagnosis but has limitations. Ultrasound has emerged as a promising tool for distinguishing between benign and malignant nodules. This study aims to compare the diagnostic accuracy of ultrasonography (USG) and FNAC in diagnosing malignant thyroid swelling using postoperative histopathological examinations as the gold standard.

Method: A diagnostic accuracy study was conducted over 1.5 years at Rajendra Institute of Medical Sciences, Ranchi, India. A total of 132 patients with thyroid swellings were included. Patients underwent USG and FNAC, and 99 patients subsequently underwent surgery and histopathological examination. Statistical analysis was performed to evaluate the performance of USG and FNAC, including sensitivity, specificity, accuracy, positive predictive value (PPV), and negative predictive value (NPV).

Results: The study encompassed 132 patients, predominantly 94 (71.21%) females. Most patients, i.e., 76 out of 132 (57.58%), were aged 30-50 years, with an average age of presentation at 41 years. Socioeconomic status revealed 120 (90.9%) belonging to Classes II and III. USG and FNAC exhibited sensitivities of 77.4% and 90.3%, specificities of 94.1% and 98.5%, and accuracies of 88.9% and 96.0%, respectively. FNAC demonstrated superior diagnostic performance metrics compared to USG, with higher PPV and NPV, indicating its stronger ability to correctly identify true-positive cases. Ultrasound features and FNAC findings showed significant associations with biopsy results, reaffirming their utility in diagnosing thyroid nodules.

Conclusion: FNAC emerged as a highly accurate diagnostic tool for distinguishing between benign and malignant thyroid nodules, outperforming USG. Understanding demographic and clinical characteristics can aid in the timely diagnosis and management of thyroid disorders. Further research is warranted to enhance diagnostic algorithms and optimize patient care in resource-constrained settings.

## Introduction

Thyroid nodules, as defined by the American Thyroid Association, are "discrete lesions within the thyroid gland, radiologically distinct from surrounding thyroid parenchyma" [1]. These nodules pose a common clinical challenge, with a prevalence ranging from 19% to 68% in the general population [2,3]. According to the Ministry of Health and Family Welfare, India (2022), the prevalence of self-reported goiter or thyroid disorder in the National Family Health Survey IV (NFHS-IV, 2015-2016) was noted to be 2.2%, increasing to 2.9% in NFHS-V (2019-2021) [4]. Among these nodules, approximately 7% to 15% are cancerous, accounting for 96% of all new endocrine cancers [5,6].

Fine-needle aspiration cytology (FNAC) stands out as one of the most accurate methods for diagnosing malignant thyroid nodules [1,7]. Studies have reported FNAC's sensitivity at 90% and specificity at 100%, with an accuracy rate of 98.88% [8]. Despite its effectiveness, FNAC presents limitations due to its invasiveness, costliness, time consumption, and challenges in accessibility, especially in benign lesions. To address these limitations, ultrasound has emerged as a promising tool for distinguishing between benign and malignant thyroid nodules.

Horvath et al. (2009) developed the Thyroid Imaging Reporting and Data System (TI-RADS), a thyroid ultrasonographic system that stratifies cancer risk into six categories based on sonographic characteristics [9]. Several studies have validated the positive results of TI-RADS in diagnosing thyroid cancer. Zhou et al. (2011) in their study on the diagnostic value of TI-RADS in thyroid nodules suggested specificity rates of 91% and sensitivity rates of 71% [10].

The TI-RADS classification, as introduced by Horvath et al., categorizes thyroid nodules into various risk levels. TI-RADS I indicates normal findings, while TI-RADS II signifies a benign condition with 0% cancer risk. TI-RADS III suggests probable benignity with less than 5% cancer risk. Moving up the scale, TI-RADS IV denotes suspicious nodules, subdivided into IVA, IVB, and IVC based on increasing cancer risk percentages. TI-RADS V indicates a high likelihood of malignancy, with over 95% cancer risk [9].

Alongside TI-RADS, other classification systems like the Bethesda Classification and Thy Classification system offer additional insights into thyroid nodules based on cytological findings [11]. The Bethesda Classification encompasses categories ranging from non-diagnostic to malignant, while the Thy Classification system further refines classifications based on cytological features, including neoplastic and non-neoplastic categories [12].

## Materials and methods

The study employed a diagnostic test of accuracy design over a 1.5-year period from September 2022 to March 2024, focusing on patients presenting with thyroid swellings at Rajendra Institute of Medical Sciences (RIMS) in Ranchi, India. Eligibility criteria were meticulously established to ensure the study's integrity. Inclusion criteria encompassed adult patients aged 18 years and above with palpable thyroid swellings who provided informed consent. Exclusion criteria were defined to exclude individuals with biopsy-proven malignancies lacking ultrasound (USG) or fine-needle aspiration cytology (FNAC) reports, those with a history of prior thyroid swelling surgery, neck radiation, undergoing chemotherapy or radiotherapy, or experiencing hemodynamic instability or critical illness during the study period. All eligible patients were included in the study, and sample collection was conducted methodically to maintain accuracy and relevance to the research objectives.

The sample size calculations for sensitivity and specificity, denoted by 𝑁𝑠𝑒 and 𝑁𝑠𝑝, respectively, are determined by the equations 𝑁𝑠𝑒=[𝑍^2 ^𝑆𝑒(1−𝑆𝑒)]/𝑑^2^∗𝑃𝑟𝑒𝑣𝑎𝑙𝑒𝑛𝑐𝑒 and 𝑁𝑠𝑝=[Z^2 ^Se(1−Se)]/​d^2^∗𝑃𝑟𝑒𝑣𝑎𝑙𝑒𝑛𝑐𝑒​, where 𝑍 equals 1.96 representing a confidence level of 95%. Sensitivity (Se) is noted as 0.71 (71%) and specificity (Sp) as 0.91 (91%) [10]. The width of the confidence interval was set at 0.20. The estimated sample size for sensitivity, based on a sensitivity of 0.71, is 𝑛=99, while for specificity, based on a specificity of 0.91, the estimated sample size is 𝑛=40. Consequently, a sample size of 99 is selected for the study.

After receiving approval from the Institutional Ethics Committee at RIMS, Ranchi (IEC Memo No.: 133/IEC/RIMS) and obtaining proper consent from participants, a total of 132 patients underwent evaluation and examination. They were advised to undergo USG of the thyroid swelling, with 121 subsequently proceeding to FNAC of the swelling. Following this initial evaluation, only 99 patients underwent surgery, with subsequent follow-up for histopathology reports. Postoperatively, the findings of USG and FNAC were compared with the gold-standard histopathological examination. Consequently, parameters such as true positive (TP), true negative (TN), positive predictive value (PPV), negative predictive value (NPV), sensitivity, and specificity of both USG and FNAC were assessed.

Statistical analysis involved the creation of a standard template in Microsoft Excel 2017 (Microsoft Corporation, USA) to input the collected data from the research study, which was then transferred to IBM SPSS Statistics for Windows, Version 25.0 (released 2017, IBM Corp., Armonk, NY) for further analysis. Quantitative data were expressed in terms of mean, median, and standard deviation, while qualitative data were expressed as rates, proportions, and percentages. Appropriate statistical tests, whether parametric or non-parametric, were utilized depending on the normality of the data. The Chi-square test was employed to assess differences between categorical variables, while the paired t-test was used to examine differences between continuous variables, provided that the data exhibited normal distribution.

## Results

The study included 132 patients, with 94 (71.21%) females and 38 (28.79%) males. The most common age group observed was 30-39 years, accounting for 40 (30.30%) individuals. Notably, the age range of 30-50 years constituted the majority of cases, with 76 (57.58%) patients. The average age across all patients was 41 years. When stratified by gender, females had a mean age of 39 years, while males had a mean age of 48 years (refer to Table 1 for detailed statistics).

**Table 1:** Distribution of age groups by gender among the study participants

Age group (years)	Female	Male	Total	%
Below 20 years	0	0	0	0
20-29	22	5	27	20.45
30-39	38	2	40	30.30
40-49	17	19	36	27.27
50-59	7	8	15	11.36
>=60	10	4	14	10.61
Total	94 (71.21%)	38 (28.79%)	132	100
Mean age	39 years	48 years	41 years	

The samples were collected from two distinct departments: the Department of General Surgery, which comprised the majority with 120 (90.91%) out of 132 patients, and the Department of Oncosurgery, representing a smaller subset with 12 (9.09%) out of 132 patients. These samples were procured from the RIMS, Ranchi (Table 2).

**Table 2:** Count of patients admitted under various departments

Count of patients admitted under various departments		
Admitted under department	Count	%
General surgery	120	90.91%
Onco-surgery	12	9.09%
Total	132	100%

The socioeconomic status of the participants was assessed using the modified Kuppuswamy scale [13], as shown in Table 3. A significant number of patients were classified into Class 2, consisting of 55 out of 132 individuals, and Class 3, encompassing 65 out of 132 individuals. Together, these two classes represented 120 patients, accounting for over 90% of the sample population.

**Table 3:** Distribution of biopsy findings across socioeconomic status based on the modified Kuppuswamy scale.

	Biopsy				
Socioeconomic status	Benign	Malignant	Not operated	Total	%
Upper	0	0	0	0	0%
Upper middle	27	12	16	55	41.67%
Lower middle	33	16	16	65	49.24%
Upper lower	8	3	1	12	9.10%
Lower	0	0	0	0	0%
Total	68	31	33	132	100%

The patients mostly presented with a chief complaint of swelling in the anterior aspect of the neck, exhibiting varying durations of symptoms. The predominant majority (95, 71.96%) reported a history of thyroid swelling persisting for less than one year. The distribution of patients based on the duration of their symptoms is elucidated in Table 4.

**Table 4:** Distribution of patients based on the duration of symptoms

Duration of symptom	Count	%
<3 month	20	15.15 %
3-6 months	48	36.36 %
6-12 months	47	35.60 %
12-24 months	14	10.61 %
>24 months	3	2.27 %
Total	132	100 %

Clinical evaluation of 132 cases unveiled that a predominant portion of patients exhibited unilateral thyroid swelling, constituting 85 (64.34%), while bilateral swelling was observed in 47 (35.66%) cases. Among the 85 instances of unilateral thyroid swelling, 53 were identified on the right side, and 32 were localized on the left side. Among the observed patients, 61 (46.21%) exhibited swelling with soft consistency, while 63 (47.73%) were characterized as firm. The remaining eight (6.06%) swellings presented with a hard consistency (Table 5).

**Table 5:** Distribution of consistency on palpation by side

	Side			
Consistency on palpation	R	B/L	L	Total
Firm	25	26	12	63 (47.73%)
Soft	26	15	20	61 (46.21%)
Hard	2	6	0	8 (6.06%)
Total	53	47	32	132

**Figure 1:**
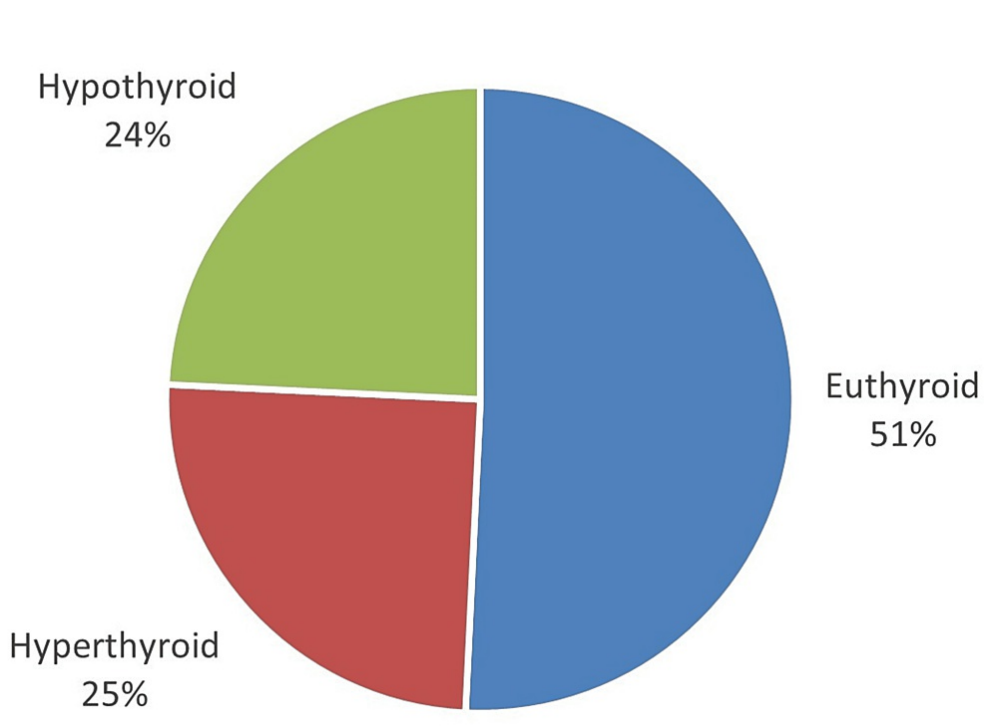
Thyroid function status among the participants.

The thyroid profile examination conducted on the patients provided significant insights into their thyroid function. The findings revealed a varied distribution of thyroid function statuses among the examined individuals. Out of the total 132 patients assessed, more than half were categorized as euthyroid, comprising 67 (50.76%) of the sample. Hyperthyroid patients accounted for 33 cases (25%) of the total, while a similar number of patients, 32 (24.24%), were diagnosed with hypothyroidism (Figure 1).

**Figure 2:**
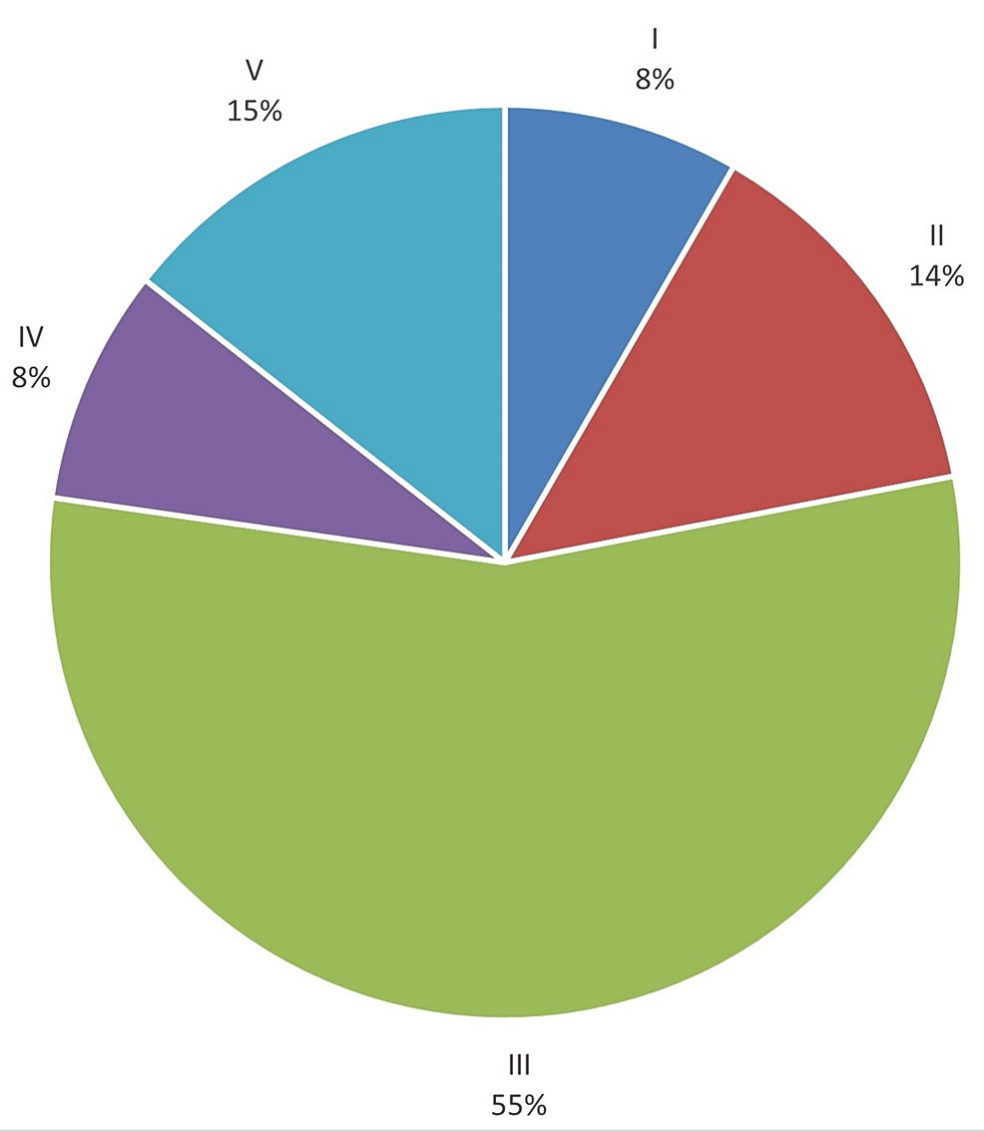
Distribution of participating patients among the TI-RADS scores TI-RADS: Thyroid Imaging Reporting and Data System

All 132 participating patients underwent ultrasound examination, wherein the composition, margin, echogenicity, shape, and echogenic foci of the thyroid gland were meticulously evaluated. The frequencies of these sonographic features are elucidated in Table 6. Subsequently, based on these sonographic characteristics, the TI-RADS scores were assigned to each patient. The distribution of patients across different TI-RADS scores is described in Figure 2 and Table 6.

**Table 6:** Comprehensive analysis of the thyroid nodule characteristics: composition, margin, echogenicity, shape, echogenic foci, TI-RADS and Bethesda classifications, and biopsy results TI-RADS: Thyroid Imaging Reporting and Data Systems, USG: ultrasonography

			Count	%
USG Features	Composition	Cystic/spongiform (0)	18	13.6 %
		Mixed cystic/solid (1)	111	84.1 %
		Solid (2)	3	2.3 %
	Margin	Lobulated/irregular (2)	42	31.8 %
		Smooth/ill-defined (0)	90	68.2 %
		Extra-thyroid extension	0	0%
	Echogenicity	Anechoic (0)	13	9.8 %
		Hyperechoic (2)	100	75.8 %
		Isoechoic (1)	18	13.6 %
		Very hyperechoic (3)	1	0.8 %
	Shape	Taller than wider (3)	13	9.8 %
		Wider than taller (0)	119	90.2 %
	Echogenic Foci	Macrocalcification (1)	19	14.4 %
		None or large comet tail artifact(0)	106	80.3 %
		Punctate echogenic foci (3)	7	5.3 %
TI-RADS classification (USG)		I benign (0 pts)	11	8.33%
		II non-suspicious (2 points)	18	13.64%
		III mildly suspicious (3 points)	73	55.30%
		IV moderately suspicious (4-6 points)	11	8.33%
		V highly suspicious (>= 7 points)	19	14.39%
		Total	132	100%
Bethesda score (FNAC)		1 non-diagnostic	2	1.65%
		2 benign	21	17.36%
		3 atypical	39	32.23%
		4 suspicious or follicular neoplasm	28	23.14%
		5 suspicious for malignancy	31	25.62%
		Total	121	100%
Biopsy	Benign	Benign nodule	8	8.08%
		Colloid goiter	39	39.39%
		Multinodular goiter	11	11.11%
		Thyroiditis	10	10.10%
		Total	68	68.68%
	Malignant	Follicular neoplasm	7	7.07%
		Papillary carcinoma	24	24.24%
		Total	31	31.31%
	Grand total		99	100%

Out of the 132 patients who underwent ultrasound examination, 121 proceeded to FNAC examination. Notably, among these, nine out of 11 patients with a TI-RADS score of 1 opted out of FNAC, along with one patient each from the TI-RADS 2 and TI-RADS 3 groups. The results of the FNAC were categorized utilizing the Bethesda scoring system. The most prevalent Bethesda score observed was Bethesda 3 (atypical), which accounted for 39 out of 121 cases, comprising 32.23% of the total. A detailed breakdown of the distribution among different Bethesda scores is outlined in Table 6.

Out of the total 132 participants, only 99 (75%) underwent surgical intervention. Among these 99 operated cases, the majority underwent total thyroidectomy, accounting for 94 (94.95%) cases, while the remaining five (5.05%) cases underwent either subtotal thyroidectomy or hemithyroidectomy. A summary of the surgical procedures performed is presented in Table 7.

**Table 7:** Distribution of surgical procedures done

Row labels	Count of procedure done (%)	%
Hemithyroidectomy/subtotal thyroidectomy	5	5.05%
Total thyroidectomy	94	94.95%
Grand total	99	100%

Following surgical intervention, all specimen tissues were subjected to histopathological examinations. Out of the 99 histopathological examinations conducted, 68 (68.68%) yielded benign results, while 31 (31.31%) were identified as malignant. Further classification of benign tissues revealed colloid goiter as the most prevalent histological diagnosis, with 39 cases. Multinodular goiter, thyroiditis, and benign nodules were observed in 11, 10, and eight cases, respectively. Among the 31 malignant tissues, papillary carcinoma was the predominant histological finding, accounting for 24 cases (77.42%), while the remaining seven cases were identified as follicular neoplasm. Details of these histopathological findings are summarized in Table 6.

Table 8 summarizes the USG features along with their respective sensitivity, specificity, PPV, NPV, and accuracy.

**Table 8:** USG features and diagnostic performance metrics USG: ultrasonography

USG features		Sensitivity	Specificity	PPV	NPV	Accuracy
Composition	Cystic/spongiform	0	92.6	0	67	63.6
	Mixed cystic/solid	96.8	10.3	33	87.5	37.4
	Solid	3.2	97.1	33.3	68.8	67.7
Margin	Lobulated/irregular	80.6	76.5	77.8	89.7	77.8
	Smooth/ill-defined	19.4	23.5	10.3	39	22.2
Echogenicity	Anechoic	6.5	91.2	25	68.1	64.6
	Hyperechoic	87.1	13.2	31.4	69.2	36.4
	Isoechoic	3.2	95.6	25	68.4	66.7
	Very hyperechoic	3.2	100	100	69.4	69.7
Shape	Taller than wider	61.3	100	100	78.2	80.8
	Wider than taller	38.7	0	21.8	0	19.2
Calcification	Macrocalcification	35.5	91.2	64.7	75.6	73.7
	None or large comet tail artifact	41.9	8.8	17.3	25	19.2
	Punctate echogenic foci	22.6	100	100	73.9	75.8

Table 9 elucidates the distribution of histopathological outcomes and Bethesda scores across distinct TI-RADS scores. All the cases with TI-RADS scores I and II exhibited benign biopsy results, devoid of any malignant findings. By contrast, TI-RADS score III predominantly showcased benign outcomes, constituting 58 out of 73 cases (79.45%), albeit with some cases demonstrating malignancy (seven out of 73, 9.59%). TI-RADS score IV displayed a heterogeneous distribution, featuring a combination of benign (four out of 11, 36.36%) and malignant (six out of 11, 54.54%) biopsy results, with certain cases not undergoing surgical intervention and biopsy. Conversely, TI-RADS score V exhibited a predominance of malignant biopsy results, encompassing 18 out of 19 cases (94.74%).

**Table 9:** Distribution of histopathological outcomes and Bethesda scores across TI-RADS scores TI-RADS: Thyroid Imaging Reporting and Data System

TI-RADS score	Biopsy	FNAC score (Bethesda scoring)						Grand total	%
		1	2	3	4	5	FNAC not done		
I	Benign	1	1	0	0	0	0	2	1.51
	Malignant	0	0	0	0	0	0	0	0
	Not done	0	0	0	0	0	9	9	6.82
Total		1	1	0	0	0	9	11	8.33
II	Benign	1	1	0	2	0	0	4	3.03
	Malignant	0	0	0	0	0	0	0	0
	Not done	0	11	2	0	0	1	14	10.61
Total		1	12	2	2	0	1	18	13.64
III	Benign	0	2	35	19	2	0	58	43.94
	Malignant	0	0	0	2	5	0	7	5.30
	Not done	0	5	2	0	0	1	8	6.06
Total		0	7	37	21	7	1	73	55.30
IV	Benign	0	0	0	4	0	0	4	3.03
	Malignant	0	0	0	1	5	0	6	4.55
	Not done	0	1	0	0	0	0	1	0.76
Total		0	1	0	5	5	0	11	8.33
V	Benign	0	0	0	0	0	0	0	0
	Malignant	0	0	0	0	18	0	18	13.64
	Not done	0	0	0	0	1	0	1	0.75
Total		0	0	0	0	19	0	19	14.39
Grand total		2 (1.51 %)	21 (15.91%)	39 (29.55%)	28 (21.21%)	31 (23.48%)	11 (8.33%)	132	100

**Figure 3:**
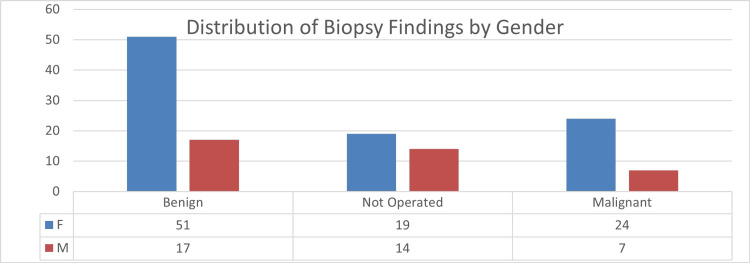
Distribution of biopsy findings by gender

The following figure presents a comprehensive breakdown of biopsy findings categorized by gender, illustrating the distribution of benign, malignant, and cases not operated (Figure 3).

The comprehensive breakdown of biopsy results categorized by age groups and gender is shown in Table 10. These data provide valuable insights into the distribution of benign and malignant biopsy outcomes across different age categories and gender demographics.

**Table 10:** Distribution of biopsy results across age groups and genders

Count of the age group		Biopsy results							
Age group (years)	Gender	Benign	Malignant	Not operated	Grand total		%		
Below 20 years		0	0	0	0		0		
20-29	F	17	3	2	22		16.67		
	M	4	0	1	5		3.78		
Total		21	3	3	27		20.45		
30-39	F	13	12	13	38		28.78		
	M	2	0	0	2		1.52		
Total		15	12	13	40		30.30		
40-49	F	12	4	1	17		12.88		
	M	5	5	9	19		14.39		
Total		17	9	10	36		27.27		
50-59	F	5	1	1	7		5.30		
	M	3	1	4	8		6.06		
Total		8	2	5	15		11.36		
60 years and above	F	4	4	2	10		7.57		
	M	3	1	0	4		3.03		
Total		7	5	2	14		10.61		
Grand Total		68 (51.51%)	31 (23.48%)	33 (25%)	132		100		

The following table represents the results of a chi-square (χ²) test examining the association between the USG findings categorized as TI-RADS scores and FNAC findings as Bethesda classification scores. The χ² value, degrees of freedom (df), and p-value are provided to evaluate the significance of the association. In this instance, the χ² value is reported as 65.2. The reported p-value is <0.001 (Table 11).

**Table 11:** Association between the FNAC findings and Bethesda classification scores χ² value: The Chi-square (χ²) value obtained from the statistical analysis, indicating the degree of association. df: degrees of freedom associated with the Chi-square test. n: The sample size used in the analysis. p-value: The probability value associated with the Chi-square test, indicating the significance level of the association. TI-RADS: Thyroid Imaging Reporting and Data System, FNAC: fine-needle aspiration cytology

USG	FNAC		Total	χ² test		Odds ratio = 56.7	
	Bethesda 5-6	Bethesda 1-4			Value	df	p
TI-RADS 4-6	24	6	30	χ²	65.2	1	<0.01
TI-RADS 1-3	6	85	91	N	121		
Total	30	91	121				

The relationship between the gold-standard biopsy results and USG findings, with a specific focus on distinguishing malignant from benign cases, is described in Table 12. It presents the observed frequencies of biopsy outcomes across different categories of USG results, accompanied by the results of a chi-square test assessing the significance of this association. In this case, the χ² value is 53.7, the p-value is <0.001, and the odds ratio is 54.85.

**Table 12:** Association between biopsy findings and USG (TI-RADS) results TI-RADS: Thyroid Imaging Reporting and Data System, USG: ultrasonography

	Biopsy			χ² tests			
TI-RADS score	Malignant	Benign	Total		Value	df	p
I benign	0	2	2	χ²	58.8	4	<0.01
II non-suspicious	0	4	4	n	99		
III mildly suspicious	7	58	65				
IV moderately suspicious	6	4	10				
V highly suspicious	18	0	18				
TI-RADS 4-6	24	4	28	χ²	53.7	1	<0.01
TI-RADS 1-3	7	64	71	n	99	Odds ratio = 54.85	

Table 12 also shows the relationship between biopsy outcomes and TI-RADS scores, providing insights into the distribution of benign and malignant findings across different TI-RADS categories. χ² tests reported the p-value as <0.01, indicating a highly significant association between the biopsy findings and TI-RADS scores.

The TI-RADS classification scheme exhibits varying performance levels, with TI-RADS V showcasing the highest sensitivity (58.1%) and specificity (100%), contrasting with the poor sensitivity and specificity of TI-RADS I and II. Further performance metrics are provided in Table 13.

**Table 13:** Performance metrics of the Thyroid Imaging Reporting and Data System (TI-RADS) categories

	TI-RADS I	TI-RADS II	TI-RADS III	TI-RADS IV	TI-RADS V
Sensitivity	0.0 %	0.0 %	22.6 %	19.4 %	58.1 %
Specificity	97.1 %	94.1 %	14.7 %	94.1 %	100.0 %
Accuracy	66.7 %	64.6 %	17.2 %	70.7 %	86.9 %
Positive predictive value	0.0 %	0.0 %	10.8 %	60.0 %	100.0 %
Negative predictive value	68.0 %	67.4 %	29.4 %	71.9 %	84.0 %

Analysis of the relationship between the biopsy outcomes and FNAC results, with a particular emphasis on distinguishing malignant from benign cases, is described in Table 14. The observed χ² value is 81.2, the p-value is <0.001, and the odds ratio calculated is 625.33.

**Table 14:** Association between biopsy findings and FNAC results FNAC: fine-needle aspiration cytology

	Biopsy			χ² tests		Odds ratio = 625.33	
FNAC	Malignant	Benign	Total		Value	df	p-value
Bethesda 5-6	28	1	29	χ²	81.2	1	<0.01
Bethesda 1-4	3	67	70	n	99		
Total	31	68	99				

**Figure 4:**
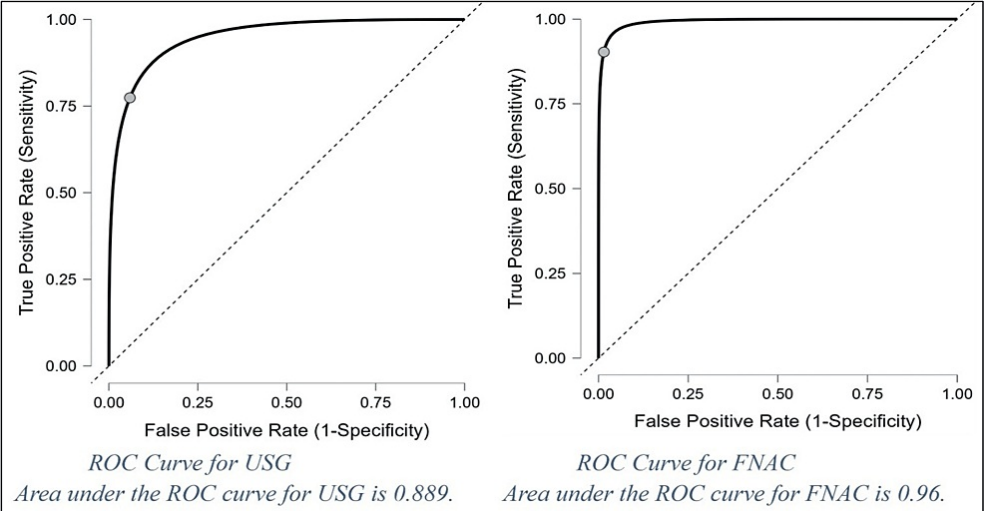
ROC analysis of TI-RADS and FNAC and cut-off values of FNAC (Bethesda categories V–VI) and USG (TI-RADS IV-VI) Accuracy is assessed using the area under the receiver operating characteristic (ROC) curve. Scores falling within the range of 0.90-1.00 are considered very good (A), 0.80-0.90 as good (B), 0.70-0.80 as fair (C), 0.60-0.70 as poor (D), and 0.50-0.60 as failing (F). ROC: receiver operating characteristic, TI-RADS: Thyroid Imaging Reporting and Data System, FNAC: fine-needle aspiration cytology

The accuracy of USG and FNAC is evaluated using ROC curves, illustrated in Figure 4.

The following table presents various performance metrics comparing USG and FNAC. These metrics provide insights into the sensitivity, specificity, accuracy, PPV, and NPV (Table 15).

**Table 15:** Comparison of the diagnostic performance metrics for USG (TI-RADS scoring) and FNAC (Bethesda scoring)

	USG (TI-RADS scoring)	FNAC (Bethesda scoring)
Sensitivity	77.4 %	90.3 %
Specificity	94.1 %	98.5 %
Accuracy	88.9 %	96.0 %
Prevalence	31.3 %	31.3 %
Positive predictive value	85.7 %	96.6 %
Negative predictive value	90.1 %	95.7 %

The distribution of benign and malignant biopsy results across different thyroid function statuses, along with the results of a chi-square (χ²) test assessing the significance of this association, is presented in Table 16.

**Table 16:** Association between biopsy findings and thyroid function

	Biopsy (benign vs. malignant)			χ² tests			
Thyroid function	Benign	Malignant	Total		Value	df	p
Euthyroid	32	15	47	χ²	2.48	2	0.29
Hyperthyroid	22	6	28	n	99		
Hypothyroid	14	10	24				
Total	68	31	99				

The distribution of biopsy findings among patients, categorized by the duration of their symptoms, is depicted in Table 17. χ² tests were conducted to examine the association between the duration of symptoms and biopsy findings, yielding a p-value of 0.491.

**Table 17:** Distribution of biopsy findings across the duration of symptoms

	Biopsy findings				χ² tests			
Duration of symptom	Benign	Malignant	Not done	Total				
<3 months	9	6	5	20		Value	df	p
3-6 months	26	9	13	48	χ²	7.43	8	0.491
6-12 months	23	10	14	47	n	132		
12-24 months	9	4	1	14				
>24 months	1	2	0	3				
Total	68	31	33	132				

## Discussion

The prevalence of thyroid nodules presents a significant clinical challenge, with a notable proportion harboring malignancy. While FNAC has conventionally been used for diagnosis, its limitations warrant the exploration of alternative approaches. USG-based systems like TI-RADS offer promising non-invasive methods for discerning benign from malignant nodules.

The demographic composition of the study revealed a predominance of middle-aged individuals, particularly in the 30-39 age bracket with a mean age of presentation of 41 years. Females were 71.21% and males were 28.79% (ratio of female:male was 2.47:1). This aligns with existing literature, which suggests that thyroid disorders often manifest during middle age, possibly due to hormonal changes or environmental factors.

In a study by Srinivas et al. (2016) [14], aimed at assessing the diagnostic accuracy of TI-RADS in distinguishing benign from malignant thyroid lesions and its role in reducing unnecessary biopsies, 343 out of 365 patients (93.97%) were female and 22 (6.03%) were male, with an average age of 33.1 years.

Similarly, Mohamed et al.'s study (2021) [15] on the efficacy of TI-RADS in evaluating thyroid neoplasms among 60 patients reported a mean age of 39.48 years, with 76.7% being female and 23.3% male. The socioeconomic distribution of participants, as assessed by the modified Kuppuswamy scale [13], shows a substantial representation from lower middle-class (49.24%) backgrounds.

A study conducted by Knudsen et al. (2003) [16], on the association of low socioeconomic status and familial occurrence of goiter with a high prevalence of goiter, also identified the social imbalance in the occurrence of goiter, which can be due to smoking habit and iodine intake.

Clinical evaluation revealed anterior neck swelling as the predominant presenting complaint, with most patients reporting symptoms persisting for less than one year. χ² tests on the duration of symptoms and biopsy findings suggested no significant association between them (p-value = 0.491).

Unilateral thyroid swelling was more prevalent, accounting for 64.39% of cases, compared to bilateral swelling, which constituted 35.61%. Among unilateral cases, the right lobe was affected in 40% of instances, while the left lobe was affected in 24.39%, with a notable predominance of firm consistency upon palpation.

In a study conducted by Sengul et al. (2020), which aimed to evaluate the association between the topographic and sonographic laterality of thyroid nodules and malignancy among 501 patients and 601 thyroid nodules, findings showed that 49.8% of nodules were in the right lobe, whereas 42.4% were on the left side [17].

Another study by Rathod et al. (2021), focusing on the clinical assessment of thyroid swelling among 50 patients, found that 54% of cases presented with bilateral lobe swelling, and 22% were confined to the right lobe [18].

Sharifi Haddad et al. (2021) conducted a study to evaluate the necessity of FNAC in thyroid nodules with a probably benign sonographic appearance among 535 patients. Results revealed that 23.7% of cases exhibited bilateral enlargement, 39.7% were in the right lobe, and 32.6% were in the left lobe [19].

Thyroid function evaluation revealed a diverse distribution of thyroid function statuses as euthyroid (50.76%) was the most common status, whereas hyperthyroid and hypothyroid cases were almost equal (25% and 24.24%, respectively). Chi-square tests were conducted to assess the relationship between thyroid status and biopsy findings, which yielded a p-value of 0.29, indicating no significant association between them.

A previous study by Kim et al. (2002) identified several USG features, including hypoechoicity, marked hypoechoicity, micro lobulated or irregular margins, microcalcification, and a taller-than-wide shape, as strong predictors of malignancy in thyroid nodules [20]. These features were also deemed suspicious for malignancy by Kwak et al. (2011) [21].

In our study, we observed that the accuracy of predicting malignancy was 67.7% for solid consistency, 77.8% for micro-lobulated or irregular margins, 69.7% for very hyperechoic nodules, 80.8% for nodules with a taller-than-wider shape, and 73.7% for nodules exhibiting microcalcification.

A study conducted by De et al. (2024) [22] further corroborated these findings. They found that USG features, such as microcalcification, were highly sensitive (80%) and specific (86.11%), while a taller-than-wider shape was highly specific (92%) but had low sensitivity (36%). Hypoechogenicity was also specific (78%) but not very sensitive (68%), and irregular margins were highly specific (89%) but not sensitive (28%) in differentiating malignant from benign thyroid nodules.

In our study, the distribution of TI-RADS scoring was as follows: 8.33% for TI-RADS 1, 13.64% for TI-RADS 2, 55.30% for TI-RADS 3, 8.33% for TI-RADS 4, and 14.39% for TI-RADS 5 categories.

Contrastingly, a study by Chandramohan et al. (2016) on the accuracy of TI-RADS in daily clinical practice reported different distributions among various scores: 19.1% for TI-RADS 2, 35.3% for TI-RADS 3, 32.1% for TI-RADS 4, and 13.6% for TI-RADS 5 categories [23].

Mondal et al. (2013) [24] and Nandedkar et al. (2018) [25] observed a high incidence of Bethesda category II lesions, possibly attributed to patients directly seeking diagnosis at tertiary care centers without prior referral, a trend also noted in the study by Anand et al. (2020) [26]. However, in our study, most thyroid swellings (32.23%) were categorized as Bethesda category 3, with category 2 lesions accounting for 17.36% of cases.

In the analysis comparing USG findings (TI-RADS score) with FNAC results (Bethesda score), an χ² value of 65.2 was obtained. The reported p-value is <0.001, signifying a highly significant association between USG findings and Bethesda classification scores.

In our study, 99 out of the 132 participants underwent histopathological examination. Among these, 68 cases (68.68%) were diagnosed as benign, while 31 cases (31.31%) were determined to be malignant. Notably, colloid goiter emerged as the prevailing benign diagnosis, observed in 39 cases, whereas papillary carcinoma constituted the predominant malignant finding, detected in 24 cases.

Anand et al. (2020), in their cytohistological study concerning thyroid swellings, reported a comparable outcome, with 70.70% of cases identified as benign and 29.29% as malignant. They similarly identified colloid nodules as the most frequent benign finding, while papillary carcinoma emerged as the most prevalent malignant diagnosis [26].

Our study, in alignment with existing literature, indicates a progressive increase in neoplasm risk from TI-RADS 1 to TI-RADS 5. Table 18 presents a comparison of the risk of neoplasm across different TI-RADS categories in our study compared to several other relevant studies, including those conducted by Horvath et al. [9], Park et al. [27], Kwak et al. [21], Poller et al. [12], Periakaruppan et al. [28], Jabar et al. [29], and De et al. [22].

**Table 18:** Risk of neoplasm (%) across TI-RADS categories in our study vs. other literature comparisons TI-RADS: Thyroid Imaging Reporting and Data System

TI-RADS category	Our study	Horvath et al. (2009) [9]	Park et al. (2009) [27]	Kwak et al. (2011) [21]	Poller et al. (2016) [12]	Periakaruppan et al. (2018) [28]	Jabar et al. (2019) [29]	De et al. (2020) [22]
I	0	0	1.8	0	NA	0	0	
II	0	0	9.6	0	<3	0	0	
III	9.58	14.1	31.1	1.5	<30	2.2	6.9	22.72
IV	54.54	45	76.8	3.9-69.3	60-80	38.5	29.2	29.16
V	94.73	89.6	100	74.9	>95	77.8	80	86.66

Our study demonstrates favorable performance metrics of TI-RADS as sensitivity (77.4%), specificity (94.1%), accuracy (88.9%), PPV (85.7%), and NPV (90.1%) compared to the other studies, indicating its robustness in thyroid nodule assessment. Comparing our study and studies conducted by Periakaruppan et al. [17] and De et al. [18]. Notably, Periakaruppan et al. [17] reported high sensitivity and specificity, while De et al.'s study [18] exhibited relatively lower specificity and accuracy values (Table 19).

**Table 19:** Comparison of TI-RADS performance metrics between our study and others

	Our study	Periakaruppan et al. (2018) [17]	De et al. (2020) [18]
Sensitivity	77.4	92.3	80
Specificity	94.1	94.15	47.2
Accuracy	88.9	93.2	61
Positive predictive value (PPV)	85.7	54.54	51.28
Negative predictive value (NPV)	90.1	99.38	77.27

In the analysis of the association between USG and biopsy, the χ² value and p-value were 53.7 and <0.001, respectively, indicating a highly significant association between biopsy findings and USG results. The odds ratio of 54.85 elucidate that patients with TI-RADS scores of 4-6 exhibit a nearly 55-fold increase in the likelihood of malignant thyroid swelling compared to those with TI-RADS scores of 1-3.

Notably, our study reveals varying levels of neoplasm risk within each Bethesda category, with percentages ranging from 0% to 90.32%. The following table (Table 20) presents a comparison of the risk of neoplasm across different Bethesda categories in our study and several other studies, including those conducted by Anand et al. [26], Yang et al. [30], Jo et al. [31], Wu et al. [32], and Park et al. [33]. Comparisons with other studies indicate consistency in some categories but disparities in others, highlighting the complexity of Bethesda classification and the need for further research and standardization in interpreting cytological findings.

**Table 20:** Comparative analysis of neoplasm risk (%) across Bethesda categories between our study versus other studies

Bethesda category	Risk of neoplasm in our study (%)	Yang et al. (2007) [30]	Jo et al. (2010) [31]	Wu et al. (2012) [32]	Park et al. (2014) [33]	Anand et al. (2020) [26]
I	0	10.9	8.9	24	35.3	0
II	0	7.3	1.1	14	5.6	14.1
III	0	13.5	17	44	69.0	100
IV	10.71	32.2	25.4	67	50	81.8
V	90.32	64.7	70	77	98.7	100

Our study demonstrates favorable diagnostic performance metrics for FNAC with Bethesda scoring, including sensitivity (90.3%), specificity (98.5%), accuracy (96.0%), PPV (96.6%), and NPV (95.7%). In comparison with other referenced studies, such as Afroze et al. (2002) [34], Bongiovanni et al. (2012) [35], Nandedkar et al. (2018) [25], De et al. (2020) [22], Mohamed et al. (2021) [15], and Grimmichova et al. (2022) [36], our investigation stands out for its competitive performance across all metrics, signifying its reliability and proficiency in thyroid nodule assessment (Table 21).

**Table 21:** Comparative analysis of fine-needle aspiration cytology (FNAC) performance using Bethesda scoring: our study versus other studies

	Our study	Afroze et al. (2002) [34]	Bongiovanni et al. (2012) [35]	Nandedkar et al. (2018) [25]	De et al. (2020) [22]	Mohamed et al. (2021) [15]	Grimmichova et al. (2022) [36]
Sensitivity	90.3 %	61.9%	97%	85.7%	80.0	72.2%	70.8%
Specificity	98.5 %	99.31%	50.7%	98.6%	90.0	81.0%	91%
Accuracy	96.0 %	94.58%	68.8%	97.7%	85.0	78.3%	81.1%
Positive predictive value	96.6 %	92.86%	55.9%	-	86.0	61.9%	-
Negative predictive value	95.7 %	94.74%	96.3%	-	86.6	87.2%	-

The χ² value observed in the analysis between FNAC and biopsy was 81.2, with a p-value of <0.001, indicating a highly significant association between biopsy findings and FNAC results. The odds ratio was calculated at 625.33, suggesting that individuals classified as Bethesda 5-6 have a 625.33 times higher risk of malignant thyroid swelling compared to those classified as Bethesda 1-4.

Accuracy was further assessed using an ROC curve analysis. The area under the ROC curve (AUC) for USG was 0.889, indicating an accuracy of 88.9%, while the AUC for FNAC was 0.96, indicating an accuracy of 96.0%.

In a study by Senghul et al. [17], AUC values ranging from 0.875 to 0.895 were reported for FNAC Bethesda scoring across different lobes of the thyroid. Grimmichova et al. (2022) [36] found AUC values of 0.682 and 0.811 for USG and FNAC, respectively, in their study. They identified cut-off values >5 for both FNAC (Bethesda category V-VI) and ACR TI-RADS (TR5).

USG has a sensitivity of 77.4%, while FNAC has a higher sensitivity of 90.3%, indicating FNAC's ability to detect true positive cases more effectively, whereas USG exhibits a specificity of 94.1%, while FNAC demonstrates a higher specificity of 98.5%, indicating FNAC's ability to correctly identify true negative cases more accurately. The accuracy of USG was 88.9%, whereas FNAC showed a higher accuracy of 96.0%, reflecting FNAC's overall correctness in diagnostic outcomes. FNAC displays higher positive and negative predictive values compared to USG, indicating FNAC's stronger ability to correctly identify true positive cases. Overall, FNAC demonstrates superior diagnostic performance across most metrics compared to USG with TI-RADS scoring.

The correlation between thyroid function and the distribution of benign and malignant biopsy results, with a p-value of 0.29 indicating no statistical significance. This finding is consistent with the results reported by Kurnia Ahmad et al. (2023) in their study investigating the correlation between thyroid hormone status and histopathological characteristics of thyroid cancer [37].

Limitations

The limitations of the study included its potential selection bias and reliance on limited sample data from a single center. In addition, variations in operator skills and equipment settings may have influenced USG and FNAC results.

## Conclusions

Our study underscores the prevalence of thyroid disorders among middle-aged individuals, with a particular emphasis on the female demographic, while also highlighting the significant impact of socioeconomic factors. Notably, we found no statistically significant correlation between thyroid function status or symptom duration and the likelihood of malignancy. However, our analysis revealed compelling associations between USG features and FNAC results with biopsy findings. Specifically, FNAC exhibited notably superior diagnostic performance metrics compared to TI-RADS. Nonetheless, we advocate for further research aimed at standardizing interpretations of cytological findings and refining diagnostic algorithms.
